# Decanoic Acid and Not Octanoic Acid Stimulates Fatty Acid Synthesis in U87MG Glioblastoma Cells: A Metabolomics Study

**DOI:** 10.3389/fnins.2020.00783

**Published:** 2020-07-23

**Authors:** Fabrizio Damiano, Giuseppe E. De Benedetto, Serena Longo, Laura Giannotti, Daniela Fico, Luisa Siculella, Anna M. Giudetti

**Affiliations:** ^1^Department of Biological and Environmental Sciences and Technologies, University of Salento, Lecce, Italy; ^2^Analytical and Isotopic Mass Spectrometry Laboratory, Department of Cultural Heritage, University of Salento, Lecce, Italy

**Keywords:** citric acid cycle, decanoic acid, lipid synthesis, metabolomics, octanoic acid

## Abstract

Medium-chain fatty acids (MCFA) are dietary components with a chain length ranging from 6 to 12 carbon atoms. MCFA can cross the blood-brain barrier and in the brain can be oxidized through mitochondrial β-oxidation. As components of ketogenic diets, MCFA have demonstrated beneficial effects on different brain diseases, such as traumatic brain injury, Alzheimer’s disease, drug-resistant epilepsy, diabetes, and cancer. Despite the interest in MCFA effects, not much information is available about MCFA metabolism in the brain. In this study, with a gas chromatography-mass spectrometry (GC-MS)-based metabolomics approach, coupled with multivariate data analyses, we followed the metabolic changes of U87MG glioblastoma cells after the addition of octanoic (C8), or decanoic (C10) acids for 24 h. Our analysis highlighted significant differences in the metabolism of U87MG cells after the addition of C8 or C10 and identified several metabolites whose amount changed between the two groups of treated cells. Overall, metabolic pathway analyses suggested the citric acid cycle, Warburg effect, glutamine/glutamate metabolism, and ketone body metabolism as pathways influenced by C8 or C10 addition to U87MG cells. Our data demonstrated that, while C8 affected mitochondrial metabolism resulting in increased ketone body production, C10 mainly influenced cytosolic pathways by stimulating fatty acid synthesis. Moreover, glutamine might be the main substrate to support fatty acids synthesis in C10-treated cells. In conclusion, we identified a metabolic signature associated with C8 or C10 addition to U87MG cells that can be used to decipher metabolic responses of glioblastoma cells to MCFA treatment.

## Introduction

Medium-chain fatty acids (MCFA), namely fatty acids with an aliphatic tail of 6–12 carbon atoms, are food constituents, extracted from some plant oils and milk, where they are mostly present in the form of triacylglycerols (Marten et al., 2006).

Unlike long-chain fatty acids (LCFA) which, after intestinal adsorption, are esterified to triacylglycerols in enterocytes, incorporated into chylomicrons and released into the lymphatic system, MCFA directly enter the portal vein from the intestinal tract as free acids (Ashbrook et al., 1972, 1975). There, partly bound to plasma albumin, MCFA are transported to the liver where they are metabolized. Therefore, dietary MCFA are not stored in esterified form in adipose tissue (Schönfeld and Wojtczak, 2016).

Once in the liver MCFA, at least those with up to C8 carbon atoms, spread through the inner mitochondrial membrane and, in the mitochondrial matrix, are activated to acyl-CoA. This characteristic further differentiates MCFA from LCFA, the latter being activated in the cytosol by acyl-CoA synthases, and requiring the carnitine shuttle to pass the inner mitochondrial membrane and enter mitochondria (Vessey et al., 1999; Boomgaarden et al., 2009).

Medium-chain fatty acids, activated inside mitochondria, are mainly used as substrates in β-oxidation and in the citric acid cycle (Schönfeld and Wojtczak, 2016). The excess of acetyl-CoA derived from β-oxidation promotes the hepatic generation of ketones (mostly acetoacetate and β-hydroxybutyrate), which are delivered as fuels to non-hepatic tissues (Bach and Babayan, 1982; Papamandjaris et al., 1998).

Data obtained in an animal model suggest that MCFA can cross the blood-brain barrier and can be oxidized by the brain through mitochondrial β-oxidation (Spector, 1998). In this way, MCFA provide both a direct and an indirect fuel source for brain cells (neurons and astrocytes) via the generation of ketone bodies which can further be used by the brain as a metabolic fuel (Nugent et al., 2014; Andersen et al., 2017; Evans et al., 2017).

As components of ketogenic diets, MCFA are believed to act as alternative energy substrates in the injured brain. Indeed, MCFA are used as alternative energy substrates in conditions of limited cerebral glucose availability, such as in traumatic brain injury (Prins and Matsumoto, 2014; Bernini et al., 2020), patients with glucose transporter 1 deficiency (Klepper et al., 2004), and Alzheimer’s disease (Rusek et al., 2019). Considering that the brain is highly susceptible to free radical formation (Romano et al., 2017), an antioxidant effect of an MCFA-based ketogenic diet on the brain has also been reported (Jarrett et al., 2008). Moreover, besides having antioxidant properties, MCFA have been used to treat a variety of disorders such as drug-resistant epilepsy (Nathan et al., 2019), diabetes (Li et al., 2020), and cancer (Weber et al., 2019).

The beneficial effects of MCFA on the brain are mainly attributed to the ability of these fatty acids to provide energy sources in the form of lactate and ketone bodies (Thevenet et al., 2016). Indeed, an *in vitro* study reported that C10, but not C8 induced energy metabolism and mitochondrial activity in the neuroblastoma cell line SH-SY5Y (Khabbush et al., 2017) and increased mitochondrial content and complex I activity in neuronal cells (Hughes et al., 2014).

Despite the relative success of MCFA in the treatment of different brain diseases, the precise mechanism of action of these fatty acids in the brain is still unknown.

In the last few years, metabolite quantification by Mass Spectrometry approaches has been used to obtain a global unbiased view of small molecules in different biological samples thus contributing to the understanding of the molecular characteristics of many diseases and therapeutic outcomes in different pathologies (Vergara et al., 2019; Casadei-Gardini et al., 2020).

In the present work, by means of a gas chromatography-mass spectrometry (GC-MS)-based metabolomics approach we revealed metabolic changes in U87MG glioblastoma cells after the addition of C8 or C10 for 24 h.

Our results underline significant differences in C8 and C10 effects on U87MG cell metabolism. In particular, while C8 addition to glioma cells mainly affects mitochondrial metabolism with increased synthesis of ketone bodies, C10 has a major effect on cytosolic pathways by increasing glucose conversion to lactate. Moreover, C10 but not C8 increases lipid synthesis as demonstrated by both metabolomic and western blot analyses.

## Materials and Methods

### Reagents

Chemicals were purchased from Sigma-Aldrich (Buchs, Switzerland) or Thermo Scientific (Reinach, Switzerland). Stocks of octanoic acid (C8; C2875; Sigma-Aldrich) and decanoic acid (C10; 21409; Sigma-Aldrich) were prepared in DMSO at 100 mM.

### Cell Culture Conditions and MTT Test

U87MG cells were cultured in low-glucose (1 *g*/Lt) Dulbecco’s modified Eagle’s medium (DMEM; Corning Inc., New York, NY, United States) supplemented with 10% (v/v) heat-inactivated fetal bovine serum (FBS), 100 units/mL penicillin and 100 μg/mL streptomycin. Cells were seeded at a density of 1 × 10^6^ cells into 75 cm^2^ flask and incubated in a humidified atmosphere (5% CO_2_) at 37°C for 24 h. Cell cultures were then incubated with 300 μM C8 or C10 for 24 h. At the end of the incubation period, the cell monolayers were washed three times with PBS, trypsinized and centrifuged at 150 *g* for 3 min. The resulting cell pellet was collected for further analysis.

MTT test was performed to assay cell viability. U87MG cells were seeded at a density of 2 × 10^5^ cells/well in a 12-well culture plate (Corning Inc.) in a DMEM medium. After 24 h, the cells were treated as indicated above and the cell monolayers were incubated for 4 h with 1 mg/ml 3-(4,5-Dimethylthiazol-2-yl)-2,5-diphenyltetrazolium bromide (MTT). Formazan crystals forming in the living cells were dissolved in 1 ml 40 mM HCl in isopropanol, and the absorbance was measured at 570 nm using a Beckman Coulter DU 800 spectrophotometer.

### Protein Content Determination

Protein content was determined with Bradford reagent (1856209; Thermo Scientific), with bovine serum albumin (BSA) as a standard.

### Metabolite Determination by Gas Chromatography Coupled to Mass Spectrometry

Metabolite analyses were carried out by Agilent GC-MS (6890N GC-5973inert MS) as described by Fiehn (2016) with slight modifications. Samples were extracted with a mixture of acetonitrile, isopropanol, and water (1 mL, 3:3:2, and v/v/v), centrifuged, and the supernatant transferred into a clean vial and dried; then it was recovered in acetonitrile/water (1 mL, 1:1, and v/v) centrifuged and a 450 μL aliquot was transferred into clean glass vials, 10 μL of 100 ng/μL norleucine solution added as an internal standard and dried. Samples were first methoximated with 10 μL of a 20 mg/ml MeOX solution in pyridine at 70°C for 90 min and derivatized with 90 μL of MTBSTFA (N-tert-butyldimethylsilyl-N-methyltrifluoroacetamide) and 10 μL of FAME C8-C24 mixture at 100°C for 60 min. After cooling the solution at room temperature for 5 min, 1 μL of the solution was injected into the GC-MS in splitless mode. Metabolites were separated on a DB1HT capillary column (30 m, inner diameter 250 μm, and film thickness 0.1 μm). The oven temperature was 50°C (2 min) 10°C/min up to 350°C (10 min). The total run time was 42 min. The mass detector was operated at 70 eV in the electron impact (EI) ionization mode. The ion source and transfer line temperatures were 280°C and 300°C, respectively. Agilent Chemstation was used to process the data and quantify metabolites.

### Rate of Total Fatty Acid Synthesis From [1-^14^C]acetate

After 24 h incubations of U87MG cells with C8 or C10, the rate of total fatty acid and cholesterol synthesis was followed by adding [1-^14^C] acetate (1.56 mCi/mmol) to cells. The reaction was stopped, after 1 h, with 1 ml of 0.5 N NaOH. Cells were scraped off the plates and transferred to test tubes, and after ethanol addition, samples were subjected to saponification. At the end of the saponification reaction, cholesterol was extracted three times with 4 ml of petroleum ether. The suspension was then acidified with 0.5 ml 7 N HCl and fatty acids were extracted three times with 4 ml of petroleum ether. After solvent evaporation, the radioactivity associated with labeled cholesterol and fatty acids was measured (Vergara et al., 2017; Giudetti et al., 2019).

### Cell Lysis and Western Blot Analysis

To prepare protein extracts, cells were lysed in RIPA buffer in the presence of a cocktail of protease inhibitors. To this end, the culture medium was removed from the cell culture plate and cells were rinsed with chilled PBS (Phosphate Buffered Saline) 1× buffer. PBS was then removed and cells were pelleted by centrifugation. Washed cellular pellet fractions were suspended in RIPA buffer. After the freezing and thawing procedure, the cells were centrifuged for 5 min at 10.000 rpm. Proteins were then subjected to SDS-PAGE and electroblotting on nitrocellulose membranes, which were probed with specific primary antibodies for citrate carrier (CiC; Abcam, rabbit 1:1000), ATP-citrate lyase (ACLY; Abcam, rabbit 1:1000), acetyl-CoA carboxylase (ACC; Cell Signaling, Rabbit 1:1000), fatty acid synthase (FAS; Cell Signaling, Rabbit 1:1000), and β-actin (Abcam, mouse 1:25000). Immunoblots were revealed by enhanced chemiluminescence reagent (Bio-Rad). Images were acquired using the VersaDoc 1000 imaging system and individual band densities were integrated by Quantity One software (BioRad).

### Statistical Analysis

Results are expressed as means ± standard error of the mean (SEM) of three different samples, each of which were analyzed twice. The comparison was made using the one-way analysis of variance (ANOVA) and Tukey *post hoc* analysis. Differences among groups were considered statistically significant when *p* < 0.05.

The metabolic profile of samples from control (CTR) and U87MG cells treated with C8 or C10 MCFA controls were analyzed by GC-MS. Multivariate statistical analysis was performed using MetaboAnalyst software (Chong et al., 2018). Unsupervised Principal Component Analysis (PCA) and Partial Least Squares Discriminant Analysis (PLS-DA) were applied to examine the intrinsic variation in the data. Two parameters, *R*^2^ and *Q*^2^, describe the goodness of the statistical models. The former (*R*^2^) explains the total variations in the data, whereas the latter (*Q*^2^, calculated via 10-fold cross-validation, CV) is an estimate of the predictive ability of the models (Del Coco et al., 2019; Casadei-Gardini et al., 2020). To better visualize data, a heat map was performed on metabolites and samples, using Euclidean for distance measure and Ward for clustering algorithm.

## Results

### Metabolomics Analysis and Metabolite Identification

We performed an overall study of the metabolite changes in U87MG cells after the addition of C8 or C10 MCFA. The concentration (300 μM) of C8 and C10 and the time (24 h) of cell incubation with the two fatty acids used in this study, did not induce significant changes in cell viability assayed by the MTT test (data not shown). Metabolites from each sample were extracted in parallel and analyzed by gas chromatography coupled to mass spectrometry (GC/MS). The chromatographic spectra were dominated by high-intensity signals of sugars and small molecules, such as branched-chain amino acids (leucine, isoleucine, and valine), aliphatic and aromatic amino acids (alanine, arginine, glutamine, glutamate, methionine, glycine, phenylalanine, and tyrosine), organic acids (lactate, pyruvate, citrate, acetoacetate, and β-hydroxybutyrate), and others. The number of detected features did not significantly change (corrected *p*-value < 0.05) among the different groups.

### Multivariate Analysis of C8 and C10 Effects on U87MG Cells

To normalize raw metabolic data, log transformation and Pareto scaling were performed before multivariate analysis. ANOVA analysis and *post hoc* Tukey’s test reported a total of 16 metabolites that significantly (*p* < 0.05) changed among the three groups of cells (Table 1). PCA was then carried out to visualize the global variation in the observations and to detect possible outliers among samples. None of the observations were removed as outliers.

**TABLE 1:** Quantitative comparison of metabolites that significantly changed among groups.

Name	*p*-value	-log10(p)	FDR	*Post-hoc* test
Malonate	3.8053E-8	7.4196	9.8937E-7	““C10””, – ““C8””; ““C10””, – ““CTR””; ““C8””, – ““CTR””,
Glutamine	3.385E-7	6.4704	1.1037E-5	““C10””, – ““C8””; ““CTR””, – ““C10””; ““CTR””, – ““C8””,
L-Threonine	7.3818E-5	4.1318	1.1037E-5	““C10””, – ““C8””; ““CTR””, – ““C10””; ““CTR””, – ““C8””,
Glucose	1.3344E-4	3.8747	0.0011342	““C10””, – ““C8””; ““C10””, – ““CTR””,
L-Leucine	3.4508E-4	3.4621	0.0023466	““C10””, – ““C8””; ““C10””, – ““CTR””; ““C8””, – ““CTR””,
Glycine	9.5248E-4	3.0211	0.0053974	““C10””, – ““C8””,; ““CTR””, – ““C8””,
L-Proline	0.0012758	2.8942	0.0061968	““CTR””, – ““C10””; ““CTR””, – ““C8””,
Lauric acid	0.0026778	2.5722	0.011381	““C8””, – ““C10””; ““C8””, – ““CTR””,
Isoleucine	0.0039307	2.4055	0.014664	““C10””, – ““C8””; ““C10””, – ““CTR””; ““C8””, – ““CTR””,
Asparagine	0.0043131	2.3652	0.014664	““CTR””, – ““C10””; ““CTR””, – ““C8””,
Citrate	0.0076955	2.1138	0.023786	““C10””, – ““C8””; ““C10””, – ““CTR””,
Oxaloacetate	0.0084317	2.0741	0.02389	““C10””, – ““C8””; ““CTR””, – ““C8””,
L-Valine	0.013754	1.8616	0.033582	““C10””, – ““C8””; ““C10””, – ““CTR””,
α-Ketobutyrate	0.013828	1.8592	0.033582	““C10””, – ““C8””; ““CTR””, – ““C8””,
α-Hydroxybutyrate	0.01611	1.7929	0.034673	““CTR””, – ““C10””; ““CTR””, – ““C8””,
Tyrosine	0.016317	1.7874	0.034673	““C10””, – ““C8””; ““CTR””, – ““C8””,

**p*-values were obtained from analysis of variance (One Way-ANOVA) with Tukey’s honestly significant difference (HSD) *post hoc* test. The level of statistical significance was at least at *p*-values < 0.05 with 95% confidence level. FDR, False discovery rate.*

**FIGURE 1:**
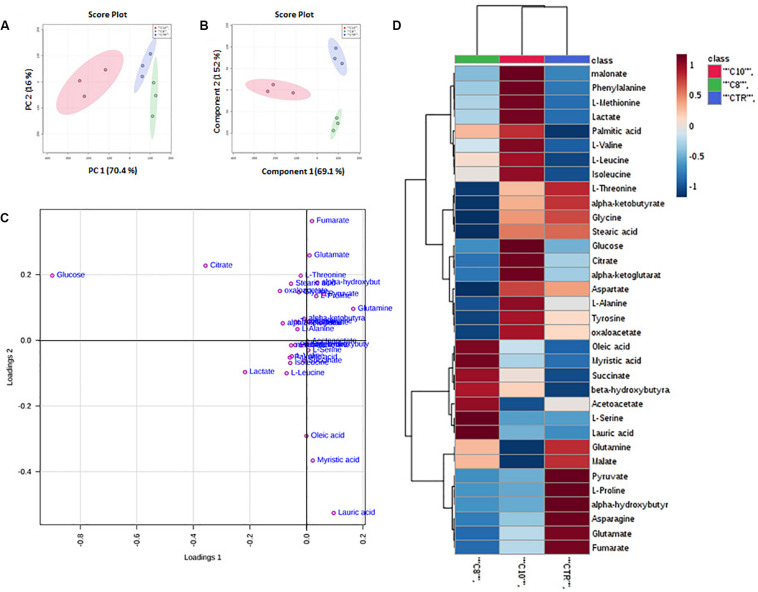
*Multivariate analysis of data from C8 or C10 treated U87MG cells*. **(A)** Principal Component Analysis (PCA) of U87MG treated with C8 or C10 (*N* = 3). **(B)** Partial Least Squares Discriminant Analysis (PLS-DA) plot. *The shaded areas indicate the 95% confidence ellipse regions based on the data points for individual groups*. **(C)** Loading plot showing the most discriminative metabolites. **(D)** Heat map visualization based on 34 metabolites. Red: C10-treated cells; green: C8-treated cells; blue: CTR cells, respectively. The color key indicates metabolite values: dark blue: lowest; dark red: highest.

Principal Component Analysis of all detected features unveiled distinct clustering of all treated groups included in the study. In particular, the groups treated with C10 clustered in the negative sector of PC1. The group treated with C8 as well as the CTR group was found in the very right sector of the PCA (Figure 1A). The PCA plot revealed a good separation especially along the first principal component PC1 (PC1 and PC2 accounted for 67.9% and 17.7% of the total variance, respectively). This result indicates that significant differences were present among the metabolomes of the treated groups.

Supervised classification was performed using the PLS-DA model to search for discriminating features for the separation among groups. A clear separation between the three groups was observed also in the corresponding PLS-DA (Figure 1B). The PLS-DA model was obtained with the first two components explaining 66.2% and 18% of the total variance.

To better understand the most discriminative metabolites among the three groups of U87MG cells, the weight of each metabolite on each component is visualized on a loading plot (Figure 1C). Metabolites that contribute more to the discrimination will have a higher weight, and thus tend to be the farthest away from the origin. As is evident, glucose, citrate, lactate, pyruvate, glutamate, glutamine, lauric, myristic, and oleic acids were, among others, the most relevant metabolites which differentiated the three groups of samples.

Moreover, a heat map of all the differential metabolites was produced to visualize the relative concentration of metabolites in each sample. On the heat map (Figure 1D), the up-regulation or down-regulation of the different metabolites was expressed with different colors, thereby the changes of the patterns in metabolite concentrations across samples are easily observed. Among others, higher levels of malonate, phenylalanine, methionine, lactate, palmitic acid, branched-chain amino acids (valine, leucine, and isoleucine) glucose, citrate, α-ketoglutarate (α-KG), alanine tyrosine and oxaloacetate (OAA), were clearly shown in C10 treated cells compared with the other groups, as well as higher levels of oleic and myristic acid, succinate, β-hydroxybutyrate, and acetoacetate were shown in C8 treated cells.

### Metabolic Pathway and Metabolite Set Enrichment Analyses

For a better understanding of metabolic pathways which can have a significant impact on a given biological process after C8 or C10 addition to U87MG cells, we performed two types of pathway analyses. Metabolite Set Enrichment Analysis, performed using the Small Molecule Pathway Database and Metabolic Pathway Analysis, performed using the KEGG database, which also calculates the impact of each pathway using topology analysis in addition to the classic Enrichment Analysis (Girelli et al., 2016; De Castro et al., 2018). According to both *p*-value and impact value, the analysis showed target pathways that could be potentially altered following C8 or C10 addition to U87MG cells.

**FIGURE 2:**
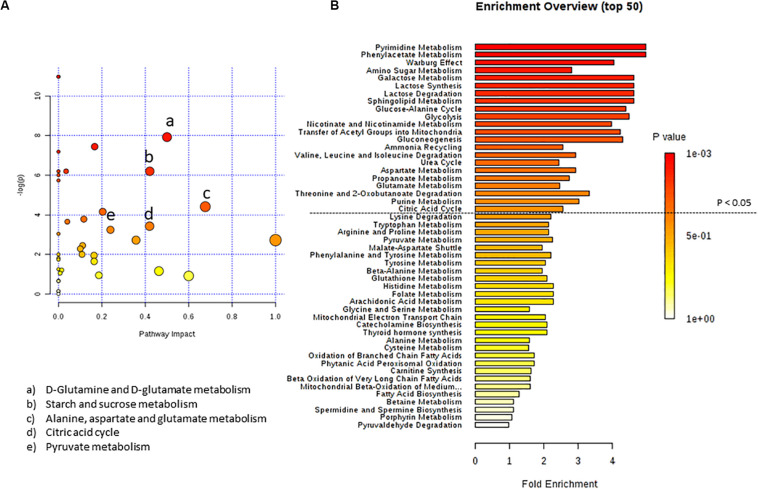
*Pathway analysis of metabolites in U87MG cells treated with C10 compared to CTR.* **(A)** Metabolomics pathway analysis using the KEGG database. **(B)** Summary plot for metabolite Enrichment Analysis performed on the U87MG cells treated with C10 vs CTR cells, where metabolite sets are ranked according to the Holm *p*-value. The horizontal bar graph summarizes metabolic pathways that were significantly different between CTR and C10-treated cells.

By inputting the metabolites that were significantly influenced by C10 addition to U87MG cells, we found matched pathways reported in Figure 2. After C10 addition to cells, the most significantly modified pathways in Metabolic Pathway Analysis (Figure 2A and Table 2) were glutamine/glutamate metabolism, starch and sucrose metabolism, alanine, aspartate, and glutamate metabolism, citric acid cycle and pyruvate metabolism. Enrichment analyses, based on the most relevant metabolites changed after C10 addition to cells, showed different metabolic pathways potentially involved after the fatty acid addition to U87MG cells, notably the Warburg effect, lactate synthesis and degradation, glucose-alanine cycle, and the citric acid cycle among others.

**TABLE 2:** Pathway analysis of metabolites from C10-treated vs CTR cells.

Pathway name	Match status	*p*-value	Impact
Purine metabolism	1/65	1.7051E-5	0.0
Pyrimidine metabolism	1/39	1.7051E-5	0.0
D-Glutamine and D-glutamate metabolism	3/6	3.6167E-4	0.5
Aminoacyl-tRNA biosynthesis	6/48	8.2099E-4	0.0
Starch and sucrose metabolism	1/18	0.0020341	0.4207
Galactose metabolism	1/27	0.0020341	0.03499
Neomycin, kanamycin and gentamicin biosynthesis	1/2	0.0020341	0.0
Nitrogen metabolism	2/6	0.0024727	0.0
Alanine, aspartate and glutamate metabolism	10/28	0.012493	0.67629
Glyoxylate and dicarboxylate metabolism	7/32	0.015648	0.13493
Arginine biosynthesis	5/14	0.022856	0.11675
Propanoate metabolism	3/23	0.026032	0.04061
Citric acid cycle	7/20	0.032615	0.41976
Pyruvate metabolism	5/22	0.039236	0.2395

*Pathways involved in C-10 treated cells with the number of metabolites that match in the indicated pathway; *p* is the original *p*-value calculated from the Enrichment Analysis; impact is the pathway impact value calculated from pathway topology analysis.*

**FIGURE 3:**
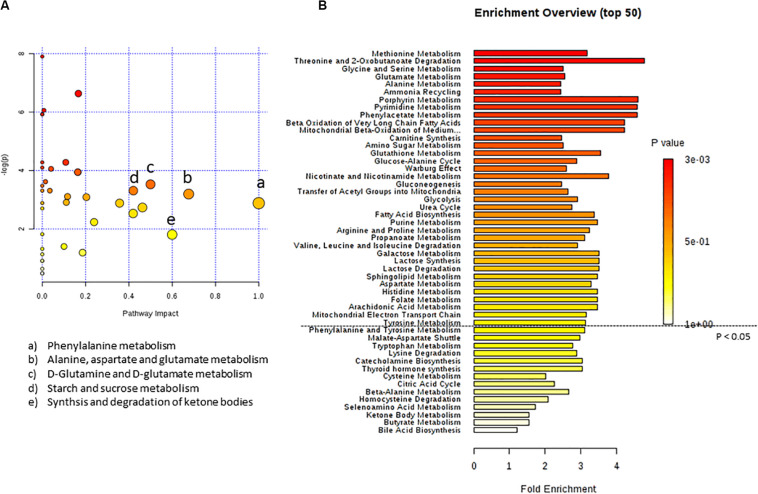
*Pathway analysis of metabolites in U87MG cells treated with C8 compared to CTR.* **(A)** Metabolomics pathway analysis using the KEGG database. **(B)** Summary plot for metabolite Enrichment Analysis performed on the U87MG cells treated with C8 vs CTR cells, where metabolite sets are ranked according to the Holm *p*-value. The horizontal bar graph summarizes metabolic pathways that were significantly different between CTR and C8-treated cells.

**FIGURE 4:**
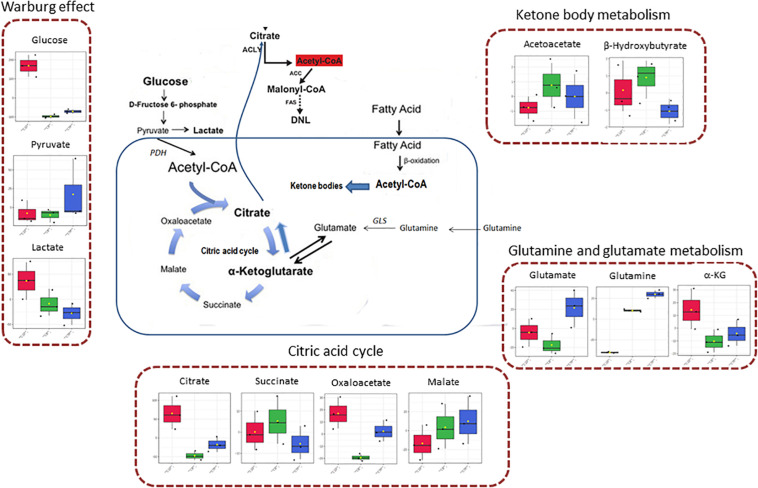
*Schematic representation of some involved pathways in C8- and C10-treated cells, on the basis of Metabolic Pathway analysis*. In the mitochondrial matrix, glutamine can be converted, through glutaminase (*GLS*) activity, in glutamate and then in α-ketoglutarate (glutamine and glutamate metabolism). In cancer cells, α-ketoglutarate can converge toward citrate that, after conversion in acetyl-CoA in the cytosol, represents the primer for lipid synthesis. α-Ketoglutarate can also generate citrate through the oxidative metabolism in the citric acid cycle, forming succinate, fumarate, malate, oxaloacetate, and citrate. Mitochondrial acetyl-CoA can derive also from fatty acids β-oxidation or glycolytic pyruvate through the activity of pyruvate dehydrogenase complex (*PDH*). When in the mitochondrial matrix acetyl-CoA exceeds OAA amounts, acetyl-CoA can be used for ketone body production (ketone body metabolism). However, cancer cells can also produce a high level of lactate from glycolytic pyruvate, also in the presence of functioning mitochondria (Warburg effect).

In Figure 3 the effect of C8 addition on cell metabolism was depicted by both analyses. In this case, major involvement of amino acid metabolism, such as phenylalanine, alanine, aspartate, glutamine, and glutamate metabolism was evident through Metabolic Pathway Analysis (Figure 3A and Table 3). Moreover, Enrichment Analysis showed that a major number of metabolic pathways were potentially involved, thus indicating a broader and less specific effect of C8 on U87MG metabolism (Figure 3B). Based on significantly changed metabolites reported in Table 1, and on Metabolic Pathway analysis of Figures 2, 3, we grouped metabolites, with the respective changes, in some pathways potentially modified after C8 or C10 addition to U87MG cells (Figure 4).

**TABLE 3:** Pathway Analysis of metabolites from C8-treated vs CTR cells.

Pathway name	Match status	*p*-value	Impact
Purine metabolism	1/65	0.0026759	0.0
Pyrimidine metabolism	1/39	0.0026759	0.0
Valine, leucine and isoleucine biosynthesis	1/8	0.0091165	0.0
Nitrogen metabolism	2/6	0.0166	0.0
Propanoate metabolism	3/23	0.017297	0.04061
Aminoacyl-tRNA biosynthesis	6/48	0.018156	0.0
Tyrosine metabolism	4/42	0.019357	0.16435
Fatty acid biosynthesis	4/47	0.021364	0.01473
D-Glutamine and D-glutamate metabolism	3/6	0.029373	0.5
Starch and sucrose metabolism	1/18	0.036503	0.4207
Galactose metabolism	1/27	0.036503	0.03499
Neomycin, kanamycin and gentamicin biosynthesis	1/2	0.036503	0.0
Glyoxylate and dicarboxylate metabolism	7/32	0.038069	0.13493
Glutathione metabolism	1/28	0.039744	0.01966
Porphyrin and chlorophyll metabolism	1/30	0.039744	0.0
Alanine, aspartate and glutamate metabolism	10/28	0.041703	0.67629
Arginine biosynthesis	5/14	0.044703	0.11675

*Pathways involved in C-8 treated cells with the number of metabolites that match in the indicated pathway; *p* is the original *p*-value calculated from the Enrichment Analysis; impact is the pathway impact value calculated from pathway topology analysis.*

### Fatty Acids Synthesis Is Increased in C10- but Not in C8-Treated Cells

**FIGURE 5:**
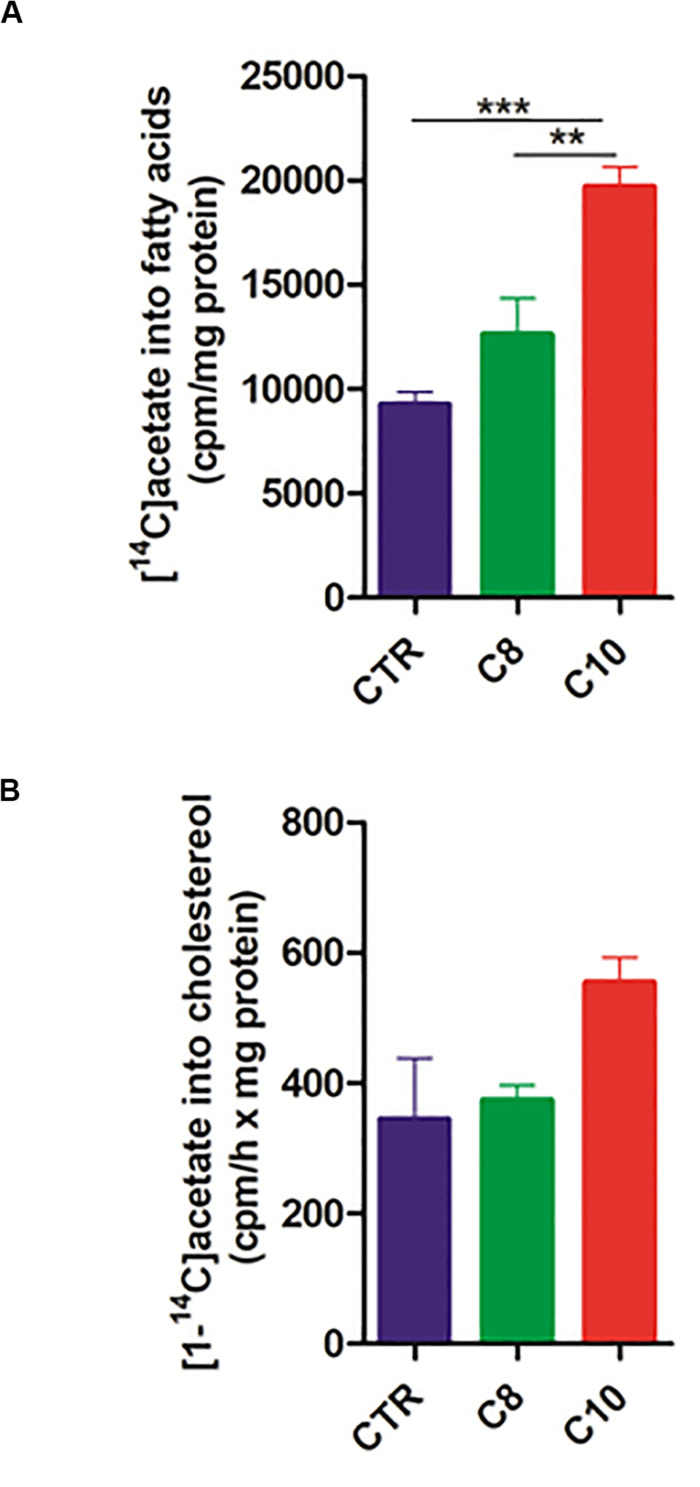
*Fatty acid and cholesterol synthesis in U87MG cells.* After 24 h incubation of U87MG cells with C8 or C10, fatty acid **(A)** and cholesterol **(B)** synthesis was measured by incubating for 1 h treated cells with [1-^14^C]acetate. The specific activity of [1-^14^C]acetate incorporation was expressed as cpm/h x mg of proteins. Values are the mean ± SEM of three different experiments **P < 0.005; ***P < 0.001.

Considering that citrate represents not only the precursor, in the form of acetyl-CoA, of fatty acid synthesis but also a positive modulator for ACC, the rate-limiting enzyme in the fatty acid synthesis pathway, we hypothesized that C10 could stimulate fatty acid synthesis in U87MG cells. To analyze the pathway of fatty acid synthesis in U87MG cells, we measured the rate of [1-^14^C] acetate incorporation into fatty acids. Interestingly, after 1 h of incubation with the labeled precursor, C10-treated cells incorporated significantly higher (∼2 fold) labeled acetate into fatty acids compared with both C8-treated and CTR cells (Figure 5A). Moreover, as acetate also represents the precursor for cholesterol synthesis, we measured the rate of cholesterol synthesis as well. As reported in Figure 5B, no significant differences were measured among groups. These data indicated that C10 specifically affected the pathway of fatty acid synthesis in U87MG cells.

**FIGURE 6:**
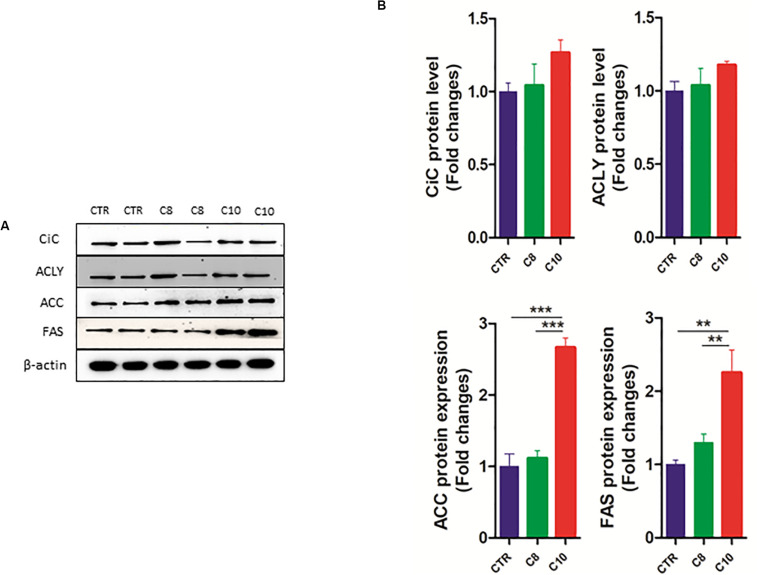
*Western blotting analysis of lipogenic enzymes*. Lysates from control (CTR) cells and from U87MG cells treated with C8 or C10 for 24 h were subjected to SDS-PAGE. **(A)** After blotting, membranes were analyzed by immunoblotting using specific antibodies for citrate carrier (CiC), ATP-citrate lyase (ACLY), acetyl-CoA carboxylase (ACC), and fatty acid synthase (FAS). β-actin was used as a loading control. **(B)** Signal intensity was quantified by densitometric analysis and reported as fold changes of C8- or C10-treated cells, compared to controls. Values are the mean ± SEM of three different experiments. **P < 0.01; ***P < 0.001.

We also measured the protein expression of CiC, ACLY, ACC, and FAS enzymes involved in the *de novo* synthesis of fatty acids. We found that the protein content of both ACC and FAS significantly increased after C10 addition, compared with both CTR and C8-treated cells (Figure 6). However, no significant changes were measured for CiC and ACLY expression among groups.

## Discussion

There is growing interest in the mode of MCFA action particularly in the brain, considering their beneficial effects on different neurological diseases that have been recently reported (Klepper et al., 2004; Nugent et al., 2014; Prins and Matsumoto, 2014; Andersen et al., 2017; Evans et al., 2017; Nathan et al., 2019; Rusek et al., 2019; Bernini et al., 2020; Li et al., 2020). Studies have shown that C8 and C10 have a different metabolism in the brain which might derive from a different cell processing of these two fatty acids. In particular, while C8 can enter mitochondria without requiring the carnitine shuttle, C10 seems to be activated in the cytosol and to require the presence of carnitine to enter mitochondria (Khabbush et al., 2017).

Although most of the beneficial effects of MCFA have been attributed to ketone body formation for a long-time, some studies have challenged the central role of these molecules, raising the question whether other possible MCFA-derived metabolites may play a relevant role in brain disease treatments.

In this study, to cover the broadest possible range of metabolic characteristics, we examined the biological effects of 300 μM C8 or C10 through an untargeted GC/MS-based metabolomics approach. It should be noted that the amount of MCFA we used throughout the study was compatible with the plasma level of these fatty acids found in epileptic patients treated with the MCFA-containing ketogenic diet (Haidukewych et al., 1982; Khabbush et al., 2017).

We found that C10-treated, but not C8-treated cells showed increased amounts of glucose and lactate compared with CTR. We speculated that C10 could increase the flux of glucose to lactate as already demonstrated for C10-treated astrocytes (Thevenet et al., 2016). This process, thought to be characteristic of cancer cells, is known as the Warburg Effect and represents the incomplete, non-oxidative metabolism of glucose even in the presence of oxygen (Warburg, 1925; Warburg, 1956). It is of note that the Enrichment Analysis highlighted a high impact of the Warburg effect, lactate synthesis and glycolysis in C10-treated cells compared with CTR.

It is well known that glucose represents the major substrate for *de novo* lipogenesis. In this case, pyruvate produced by glycolysis can enter mitochondria to be converted into acetyl-CoA by the complex of the pyruvate dehydrogenase. Acetyl-CoA, in the presence of OAA and citrate synthase enzyme, is converted into citrate that, when in excess, can be transported by CiC, a transporter of the inner mitochondrial membrane, in the cytosol to support lipid synthesis (Siculella et al., 2006; Giudetti et al., 2016). Then cytosolic citrate is converted to acetyl-CoA by ATP citrate lyase (Browning and Horton, 2004). *De novo* synthesis of fatty acids begins with ATP-dependent carboxylation of acetyl-CoA to malonyl-CoA by ACC. Malonyl-CoA, which serves as a two-carbon donor, is added to the acetyl-CoA primer by a multifunctional enzyme complex of FAS to form palmitic acid.

Citrate, in the cytosol, represents not only the primer for the synthesis of lipids but also a positive allosteric modulator for ACC, the rate-limiting enzyme in the lipogenic pathway.

Considering the higher level of citrate and palmitate measured in U87MG cells after C10-, but not C8-addition to cells, compared with CTR cells, we speculated that C10 could stimulate fatty acid synthesis in U87MG cells. It should be noted that a lipogenic effect of MCFA has been already demonstrated in isolated hepatocytes, with a greater stimulatory effect of C10 with respect to C8, reaching the highest value with C12:0 (Geelen, 1994). The higher rate of acetate incorporation into fatty acids associated with increased expression of ACC and FAS confirmed our hypothesis about the stimulated fatty acid synthesis in U87MG cells after C10- but not C8-addition.

It has been demonstrated that decanoic acid is a PPARγ agonist and this characteristic seems to be specific to decanoic acid and unlikely to be shared by octanoic acid, the other major component of the MCT ketogenic diet (Malapaka et al., 2012). Considering that PPARγ is able to regulate the lipogenic transcription factor SREBP1c, which in turn induces the expression of the lipogenic enzyme fatty acid synthase, we can speculate that C10 stimulates *de novo* lipogenesis in U87MG cells by a PPARγ-mediated mechanism (Schadinger et al., 2005). In particular conditions cancer cells prefer glutamine to glucose for lipid synthesis (Bott et al., 2019). In this case, glutamine can be converted to glutamate and α-KG in mitochondria (Bott et al., 2019). The glutamine-derived α-KG is converted to citrate through both reductive and oxidative carboxylation and thereby contributes to *de novo* lipogenesis (Bott et al., 2019). In C10-treated cells, we found an increasing amount of α-KG and citrate, with a lower level of glutamine, compared with both C8-treated and CTR cells. We speculated that C10 can increase glutamine conversion into lipids. Notably, both Metabolic Pathway analysis, and Pathway Enrichment Analysis reported an important impact of glutamine and glutamate metabolism’ on C10-treated cells.

An interesting aspect worthy of attention is the high content of malonate found in C10-treated cells compared with C8-treated and CTR cells. Malonate is an endogenous metabolite and a well-characterized competitive inhibitor of mitochondrial succinate dehydrogenase, an enzyme of the electron chain transport complex II. Malonate can be cytotoxic by blocking the citric acid cycle and cellular respiration (Bowman et al., 2017). The molecular source of malonate in eukaryotic cells is not well defined. Mitochondrial malonate may derive from the hydrolysis of mitochondrial OAA through the decarboxylase activity of OAA decarboxylase (Weiss et al., 2018). Malonate can be also formed by cytosolic malonyl-CoA hydrolysis and subsequently transported into the mitochondrial matrix via dicarboxylic acid transporters of the inner mitochondrial membrane (Bowman et al., 2017). Thus, by blocking the citric acid cycle, malonate produced in C10-treated cells could address metabolite flux vs citrate. Due to the lower level of glutamine, and the higher amount of α-KG and citrate measured in C10-treated cells compared with CTR, we speculated that C10, the increased level of malonate, upon C10 addition to the cells, could direct glutamine toward fatty acid synthesis, thus driving reductive metabolism. If this is the case, our results could explain why mitochondrial C10 βoxidation was found markedly lower than that of C8 in both SH−SY5Y cells and astrocytes (Thevenet et al., 2016; Khabbush et al., 2017).

We observed that the addition of C8 to cells increased lauric acid, myristic acid, oleic acid, β-hydroxybutyrate, and acetoacetate levels compared with both CTR and C10-treated cells. These data could indicate that C8, once activated inside the mitochondrial matrix, could undergo chain elongation before being oxidized. Hence, considering the lower level of OAA measured in C8 vs both CTR and C10-treated cells we can speculate that the acetyl-CoA derived from fatty acid oxidation could diverge toward ketone body synthesis, thus justifying the higher level of β-hydroxybutyrate and acetoacetate in C8- compared with both C10-treated cells and CTR cells. Our data are in accordance with those reported in Thevenet et al. (2016), Sonnay et al. (2019) about the increased secretion of ketone bodies after C8- more than C10-addition to astrocytes. It should be noted that the Metabolic Pathway analysis indicates the synthesis and degradation of ketone bodies as a relevant pathway in C8-treated U87MG cells. Particularly, it has been reported that a carbon chain-length elongation process precedes the oxidation of medium-chain fatty acids in skin fibroblasts (Jones et al., 2006).

Overall, our study demonstrated, for the first time, a lipogenic effect of C10 on glioblastoma cells. These data could be of interest considering that fatty acid synthesis can sustain cell proliferation, being lipid building blocks for membrane construction. On the other hand, fatty acids can cause death in cancer cells due to detergent-like effects (Leaver et al., 2002). Thus, although our study requires further consideration, it provides interesting insights and directs our attention to the potential use of decanoate in brain cancers.

## Data Availability Statement

The raw data supporting the conclusions of this article will be made available by the authors, without undue reservation.

## Author Contributions

All authors have read and agreed to the published version of the manuscript. FD and AG: conceptualization. GD and DF: methodology. SL and LG: software. AG, FD, and LS: writing – review and editing.

## Conflict of Interest

The authors declare that the research was conducted in the absence of any commercial or financial relationships that could be construed as a potential conflict of interest.
